# From loss to gain: role for SUN1 in laminopathies

**DOI:** 10.1186/2045-3701-2-21

**Published:** 2012-06-18

**Authors:** Baohua Liu, Dong-Yan Jin, Zhongjun Zhou

**Affiliations:** 1 Department of Biochemistry, The University of Hong Kong, 3/F Laboratory Block, 21 Sassoon Road, Pokfulam, Hong Kong

## Abstract

Mutations in *LMNA* encoding lamins A and C are associated with at least 10 different degenerative disorders affecting diverse tissues, collectively called laminopathies. A recent study showed that mis-accumulation of SUN1 underlies the pathology of degenerative features in laminopathies, and concomitantly suggests a gain-of-function versus a loss-of-function model for the action of lamin A mutants.

## 

The nuclear envelope (NE) is a double-layered membrane separating the cell’s genetic material from the cytoplasm. The outer nuclear membrane (ONM) fuses with the inner nuclear membrane (INM) around the nuclear pore complex and extends to the endoplasmic reticulum (ER). Right underneath the INM is a layer of fine meshwork, namely nuclear lamina wherein various lamins and interacting proteins reside. A group of SUN (Sad1/UNC-84 homology) domain-containing proteins anchor to the INM and interact with different lamins, on the lamina, and nesprins, on the ONM, thus bridging nucleoskeleton and cytoskeleton. As one of the major components of the nuclear lamina (Figure 1), lamin A is firstly synthesized as prelamin A with an extra 18 amino acids at the C-terminus, which undergo transient isoprenylation, methylation, and these residues are finally removed by proteolytic cleavage [1]. To date, more than 237 *LMNA* mutations, leading to at least 163 protein variants, are known to be associated with at least 10 different degenerative disorders, collectively referred to as laminopathy. Some of these degenerative features are recapitulated in mice deficient for *Lmna* or harboring various *Lmna* mutations. Homozygous *Lmna*^*L530P/L530P*^ mutation (*Lmna*∆9 or Lamin A∆Exon9) or loss of prelamin A-processing metalloproteinase Zmpste24 in mice phenocopies many of the progeroid features observed in Hutchinson-Gilford progeria syndrome (HGPS), one of the most severe forms of laminopathy that are predominantly caused by a 50-amino-acid deletion in prelamin A (lamin A∆50 or progerin). Currently, the phenotypes in various mouse models and human diseases are thought to be attributable to either loss or gain of function of lamin A mutants.

**Figure 1:**
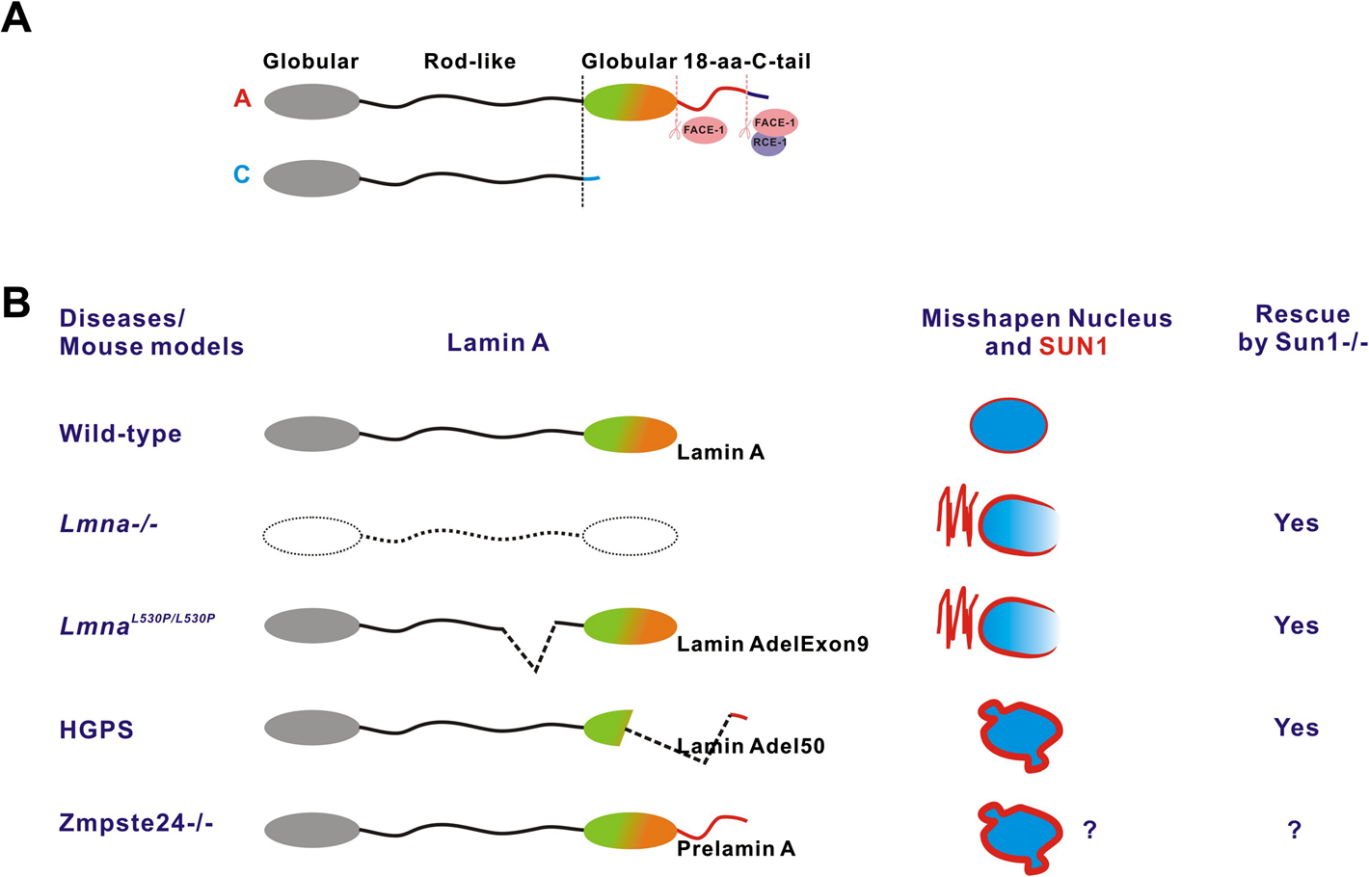
** Summary of different lamin A mutants in human diseases and mouse models. A**, Schematic diagram of lamin A/C protein and post-translational processing of prelamin A. **B**, Status of lamin A and localization of SUN1 found in mouse models and human diseases.

A recent paper further highlights the gain-of-function versus loss-of-function model for the action of lamin A in laminopathies [2]. Chen *et al.* found that the level of Sun1 protein was up-regulated and mislocalized to Golgi in *Lmna−/−* and *Lmna*∆9 cells. Although Golgi accumulation of SUN1 was not observed in HGPS cells, the levels of total and INM-localized SUN1 were significantly up-regulated and well correlated with the misshapen nucleus, one of the hallmarks of HGPS fibroblasts and cells from mouse HGPS models [3,4]. SUN proteins are major components of the LINC (Linker of Nucleoskeleton and Cytoskeleton) complex, which links the nucleoskeleton and cytoskeleton, and mediates nucleus positioning. Further investigation showed that either increased INM-association of SUN1 in HGPS or its accumulation at Golgi in *Lmna−/−* and *Lmna*∆9 cells was detrimental, as knocking down or genetically depleting SUN1 from the mutant cells largely rescued the misshapen nucleus and increased cell survival. Although all three mutant cells exhibited altered nuclear shape, they were virtually different. While loss of lamin A leads to partial loss of lamin B1 staining on NE, progerin only caused nuclear herniation in HGPS (see Figure 1). Consistent with this notion, Sun1 accumulated in the Golgi apparatus in *Lmna−/−* cells, but was properly localized on the NE in HGPS cells. In addition, while the majority of ectopic Sun1 can successfully anchor to NE, causing nucleus herniations, ectopic Golgi-orientated Sun1 led to partial loss of NE on the opposing site to the Golgi, mimicking *Lmna* null situation, in wild-type cells. Surprisingly, blocking SUN1 transportation from ER or NE towards Golgi by different small molecules restored the NE localization of both SUN1 and lamin B1. These data suggest that although lamin A is not required for the NE positioning of SUN1, it may facilitate the retention of SUN1 on NE [5] and thus prevent SUN1 from accumulation at Golgi. Chen *et al.* and others also showed that farnesylated progerin/prelamin A increased the binding capacity to SUN1 [2,5]. In HGPS, it has been proposed that altered nuclear shape (herniation) is attributed to the accumulation of farnesylated progerin on the nuclear lamina as it can be rescued via treatment with farnesyl transferase inhibitors (FTIs); the rescue of the misshapen nucleus by reducing prelamin A or progerin from the nuclear lamina ameliorates senescence in HGPS cells [6,7] and progeroid features in progeria mouse models [7-9]. Remarkably, depleting SUN1 increased life spans in *Lmna−/−* and *Lmna*∆9 mice and knocking down SUN1 in HGPS cells rescued heterochromatin loss and accelerated senescence.

The work by Chen *et al.* implicates a novel molecular mechanism for the pathogenesis of laminopathies; meanwhile it also raises many unanswered questions. Why does loss of lamin A delay the turnover of SUN1? What is the mechanism for the shuffling of SUN1 between Golgi and ER or NE? How does Golgi-accumulated SUN1 elicit degenerative phenotypes in tested mouse models? Why was Golgi-accumulated SUN1 not observed in HGPS cells? Answering these questions would help to better understand the molecular mechanism through which laminopathies are rescued by SUN1 depletion. The authors reasoned that the lacking Golgi-accumulated SUN1 in HGPS cells was attributable to a negative selection. Considering the loss-of-function versus gain-of-function model of lamin A proteins, there might be a second possibility. Given that the proper INM versus Golgi accumulation of SUN1 requires interaction with lamin A and increased binding capacity of SUN1 to farnesylated prelamin A, it is plausible to speculate that whereas loss of function of lamin A in *Lmna−/−* (loss of full-length lamin A), Lmna∆9 (loss of exon 9 of lamin A) and HGPS (loss of 50 amino acids in one allele of *LMNA*) upregulates the level of SUN1 but loses the capacity to maintain its NE-anchorage; gain of function of progerin (lamin A∆50, gain of the farnesyl C-tail) attracts more SUN1 to the NE and leads to nucleus herniation. There could be a balance between loss and gain of function of progerin, and it would be worthwhile to examine if FTIs could remove SUN1 from the NE and whether this underlies the rescuing effect of FTIs in HGPS. To further test this hypothesis, it would be of interest to determine the level of Sun1 in Zmpste24−/− mice, as well as cells ectopically expressing progerin. If this is true, one would expect no abnormal Golgi-accumulation of SUN1 but increased NE-associated Sun1 in Zmpste24 null mice due to the presence of prelamin A protein with a farnesyl C-tail. In fact, it has been recently shown that, while both prelamin A and progerin exhibited increased binding capacity to histone H3 peptide in comparison with lamin A, probably attributable to their gain of function, they showed differences in binding to H3K27 peptide [10]. On the other hand, prelamin A and lamin A had similar binding capacity to H3K27 peptide, whereas progerin had a reduced binding capacity, possibly due to a loss of function. It was proposed that these functional variations are likely attributable to structural differences, i.e. whereas both prelamin A and progerin contain an extra farnesylated and methylated C-terminal tail, the 50-amino-acids deletion differentiates progerin from prelamin A.

## Competing interests

The authors declare that they have no competing interests.

## Authors’ contributions

BL, DYJ and ZZ drafted the manuscript. All authors read and approved the final manuscript.
